# 

**Published:** 1849-04

**Authors:** 


### RESOLUTIONS OF THE DENTAL CLASS OF THE OHIO COLLEGE OF DENTAL SURGERY, 1849.

CINCINNATI, OHIO,
February, 10, 1849.

*Messrs. Editors:*

AGREEABLE to a resolution, I herewith inclose you a copy of the proceedings of the Dental Class, which you will have the kindness to publish in the Register, and confer an especial favor upon the class and a benefit to the profession, which is so nobly struggling to contribute its share of usefulness to mankind.

Most respectfully,

JNO. L. MILTON, *Sec'y.*

At a meeting of the students of the Ohio College of Dental Surgery, A. S. Dwyer was called to the Chair, and J. L. Milton appointed Secretary.

Being called upon, the Chairman stated, the object of the meeting was to express our high appreciation of the merits of the Faculty, and the facilities here presented for the acquirement of thorough theoretical and practical knowledge of dental surgery.

On motion, T. R. Willard, M. N. Manlove, and J. L. Milton, were appointed a committee to draft resolutions expressive of the object of the meeting, who reported the following, which were unanimously adopted:

*Resolved*, That the Ohio College of Dental Surgery, as at present organized, meets our most sanguine expectations and hearty approval, and that our own experience demonstrates to us the capability of the teachers, in their different departments, to impart a thorough course of instruction.

*Resolved*, That we heartily commend them to the profession and the public, as eminently proficient and untiring teachers, and in every way worthy the position they occupy.

*Resolved*, That our gratitude is especially due for the ample means of instruction we have been permitted to enjoy, in the removal of tumours, operations for diseased antrum, inserting of partial and full setts of teeth, extracting, filling, and the various operations pertaining to the profession, which we have met with in the well patronized and well managed infirmary connected with the institution, and that we regard an infirmary as a matter of high importance to the dental student.

*Resolved*, That we hereby tender our sincere thanks to the several teachers for their uniform kindness and untiring zeal in promoting our welfare, and we truly hope that they may long be spared to enjoy the brightening prospects of the institution, and see the profession, for which they are so nobly struggling, placed upon the proud eminence to which it is intrinsically entitled.

*Resolved*, That a copy of the proceedings of this meeting, signed by the Chairman and Secretary, be furnished the editor of the Dental Register of the West, and he be requested to publish the same, and also a copy, signed by the class, be presented to each of our teachers.

A. S. DWYER, *Ch'n.*

JNO. L. MILTON, *Sec'y.*

---

### STUDENTS AND GRADUATES OF THE OHIO COLLEGE OF DENTAL SURGERY.

WE hear so frequently of persons claiming to be students from the the Ohio College of Dental Surgery, and graduates of the same, that we are induced to give in the Register a list of those who have attended one or more full courses in the institution, and also the names of those who have passed their examination and received a degree.

It may be proper to remark, that the first session no regulation had been adopted by which all the tickets must necessarily be taken by each student; and hence the names of some twelve or fifteen, who took only a portion of the tickets, are omitted. For the four past years the class has averaged from twelve to fifteen, and the regular graduates (without any reference to honorary degrees) range from three to four.

The last session the class, with the exception of one, was made up with first course students; this, which made the last graduating class small, will, however, swell the next, in all probability, to triple the usual number.

LIST OF GRADUATES.

A. M. Leslie,
A. S. Talbert,
A. Berry,
B. A. Satterthwaite,
W. B. Ross,
W. R. Smith,
J. A. Weaver,
B. J. Dudley,
S. J. King,
G, L. Van Emon,
Eri Locke,
Thos. R. Willard,
A. S. Dwyer,
E. Bray.

THOSE WHO HAVE NOT YET GRADUATED, OR, IF SO, NOT IN THE OHIO COLLEGE.

Jacob A. Cook,
John J. Adair,
Clifton Branham,
H. J. Richards,
Jacob Straus,
J. C. Darragh,
J. C. McCullough,
S. D. Muse,
Henry Ahl,
W. C. Thompson,
Amos Pratt,
A. E. Boulten,
W. C. Thompson,
J. C. Weaver,
Wm. H. King,
Josiah Ramsey,
Samuel V. Hunter,
M. N. Manlove,
J. Taft,
J. L. Milton,
N. Y. Prestley.

JAS. TAYLOR, *Dean.*

